# Stakeholder Power Analysis of the Facilitators and Barriers for Telehealth Solution Implementation in China: A Qualitative Study of Individual Users in Beijing and Interviews With Institutional Stakeholders

**DOI:** 10.2196/19448

**Published:** 2022-01-19

**Authors:** Nuoya Chen

**Affiliations:** 1 Faculty of Global Studies, Justice and Rights University of Macerata Macerata Italy

**Keywords:** aging, telehealth implementation, stakeholder mapping and power analysis

## Abstract

**Background:**

Facing COVID 19, the use of telehealth solutions grows exponentially. However, despite the large investments made into telehealth solutions, the implementation process remains slow and sluggish. Moreover, during COVID-19, older people experienced difficulties and had the highest mortality rates, and those lucky enough to survive faced tremendous pressure to use QR code-based health monitoring systems.

**Objective:**

This paper aims to determine the barriers and incentives for the implementation of telehealth solutions via a case study about telehealth implementation in China.

**Methods:**

We conducted 8 semi-structured interviews following the design of the interactive learning framework (research question defining, participant recruitment, exploratory stage, consultation stage, integration stage, and follow-up interview). One interview with a government official from the National Health Commission and another interview with a government official from the China Center for Disease Control and Prevention was conducted in the exploratory stage. The consultation stage comprised one interview with a business manager from the Huawei Wearable Unit, one interview with a business manager from Alibaba Health Brain Unit, and one interview with a business manager from Xiaomi. Two interviews with doctors from Fudan University-affiliated Huashan Hospital and Fudan University-affiliated Zhongshan hospital were conducted in the integration stage. In addition, 8 focus group studies with 64 participants from rural and urban Beijing were conducted. Finally, another telephone interview with a business manager of the Xiaomi Wearable Unit was conducted in the follow-up stage.

**Results:**

Telehealth solutions are designed to assist health care providers in realizing the quadruple aim of better health outcomes, lowering health care costs, improved health care quality, and improved doctor and patient experiences. Governments have high incentives to improve health care efficiency via telehealth solutions. However, they have limited resources to make the necessary infrastructure transformation.

**Conclusions:**

To fully realize the potential of telehealth devices, heavy infrastructure investment in the telecommunication network is required beforehand to resolve the interoperability issue occurring during the data collection process for telehealth solutions. The industry also demands a mature business model incorporating collaboration between various stakeholders and industrial partners to invest in infrastructure. Governments have high interest and significant influence on building the necessary infrastructure for telehealth solution implementation in China. Industrial actors have a high interest and a medium level of power for telehealth solution implementation. Users have high interest but a lower level of power for the usage of telehealth solutions, and doctors have low interest and a medium level of power for telehealth solutions implementation.

## Introduction

### Background

COVID-19 has posed great challenges for unprepared public health care systems with an aging population. The sad truth seems to be countries with an older population with preexisting chronic disease conditions (eg, hypertension, cardiovascular disease, and diabetes) such as Italy, Spain, the Netherlands, the United Kingdom reported higher mortality rates than countries with a younger population, such as China [1,2]. The poor and older people were the proportion of the population hardest hit by the pandemic. Public health care systems lacking hospital beds, intensive care units, and qualified trained medical staff can use remote monitoring solutions to resolve the challenges encountered in combating COVID-19 [3]. Remote monitoring solutions can potentially improve prevention, diagnosis, treatment, and recovery efficiency by promoting data accuracy [4].

The World Economic Forum has listed the 7 biggest breakthroughs in the health care industry with “Artificial intelligence (AI) can detect skin cancer better than a doctor,” and “your phone will know if you are depressed or not” ranked 3rd and 4th on the list [5]. Exciting innovations in the health care industry, including utilizing telemedicine to transfer care to the home setting, using AI to reduce physician workload and divert patients to the right doctor, and the internet of things (IoT) to improve patient monitoring and coaching have signaled the health care industry is on the cusp of an AI revolution. Huge amounts of investment made by the public and private sectors have poured into big data, cloud computing, and utilizing such tools in the health care continuum. AI promises high-value health care and the potential to achieve the impossible health care trinity of access, quality, and cost. This paper aims to determine whether AI has achieved such an aim using empirical evidence from China.

### Concept Development

#### The Use of AI and IoT in Health Care

COVID-19 had pushed the digitalization of the health care system. As a result, investments in health-related projects have grown rapidly. For example, AI has been deployed to detect disease concentration and spread, provide real-time monitoring, and predict pandemic outbreaks and mortality risk [6]. AI has also been instrumental in COVID-19 diagnosis by performing image recognition for x-ray and magnetic resonance imaging results. For hospital management, AI has become useful in facilitating resource allocation by automizing resource management and supply chain management, assisting staff training with virtual reality (VR) and augmented reality tools, maintaining health care records, and identifying patterns for trend recognition [6].

In pandemic tracking and prediction, AI has been useful for collecting social media data and identifying disease clusters. At the beginning of the pandemic, Bluedot reported the disease cluster of pneumonia cases in Wuhan by analyzing news reports on December 31st, 2019, well ahead of public health administrations in China and other economies [7]. The Johns Hopkins University Coronavirus Resource Centre collects publicly available information and visualizes the data, making it possible to actively track the spread of the disease [8]. It is now possible to use Google Maps to estimate active COVID-19 cases by country [9].

In contact tracing, US universities such as MIT and Harvard University have been developing and using contact-tracing apps such as Safe Paths [10]. Tech companies such as Google and Apple are also working together to develop contact tracing application programming interfaces (Apple, 2020). In addition, mobile apps were developed quickly by different governments and tech companies to facilitate contact tracing in China (WeChat) and the Netherlands (Coronamelder). For example, apps such as AI4COVID-19 have enabled COVID-19 detection based on 3 seconds of coughing and delivering the diagnosis with 2 minutes [11].

During the early diagnosis of COVID-19, algorithms were rapidly developed and deployed by tech companies in China to identify patients with COVID-19 symptoms. After the Chinese New Year, where large-scale infections were reported, Yitu Technology developed the algorithms to facilitate diagnosis and treatment [12]. The software quickly received clinical approval and was deployed in Hubei and then nationally within health care systems hardest hit by the pandemic. The AI assistant received approval from the health care service staff. Congestion in hospitals was relieved with the deployment of such systems, with patients diverted to infectious disease hospitals. Yitu aims to establish the AI-assisted paradigm in four stages of the pandemic control process. In the prevention stage, chatbots and online consultation can educate users and help users perform self-examinations. In the quarantine stage, the system can help doctors monitor patients and manage their conditions.

Regarding patient management, AI has been deployed at hospitals to automate asset management [13] and prioritize COVID-19 patients in intensive care units for access to ventilators. AI can also predict the possibility of patient recovery and mortality by monitoring patients’ daily electronic health records and helping doctors to make decisions regarding the subsequent treatment steps [6].

In pharmaceutical development, AI can accelerate drug and vaccination discovery by reducing the time for drug discovery, virtual screening, and validation [6]. As a result, researchers have quickly obtained genetic information from patients and offered it to the international community [14]. In addition, AI has made it easier to predict the protein structure [15], allowing pharmaceutical companies to rapidly develop the vaccination for COVID-19. For instance, AI has been used to develop the messenger RNA vaccine by Oxford University and Moderna [16].

#### Stakeholder Analysis for the Implementation of Telehealth Solutions in Developing Economies: The Case of South Africa

The implementation of telehealth solutions in low-income economies may encounter different challenges compared to high-income economies. Therefore, when analyzing the available literature, it is necessary to address the challenges posed by the health care system in low-income economies. This paragraph considers, as an example, the case of South Africa.

Lack of infrastructure and trained medical care staff [17-19] has been known to be a hurdle to providing health care services in low-income economies. However, telehealth solutions can become a means for providing health interventions [20-23], preventing communicable disease, and improving the health literacy of health care workers and patients.

The author used a meta-study methodology and surveyed 108 papers to analyze the interactions between different stakeholders for implementing telehealth solutions in low-income economies [24]. Overall, 65% of all sample papers are from African countries. Around 26% of the sample papers come from Asia. Most of the initiatives in Africa are funded by public-private partnerships, nongovernmental organizations, or overseas initiatives [25]. The stakeholders are organized into five categories: patients, health care workers, facilitators, knowledge base, and system developers. Each stakeholder's perspective was then investigated for the interaction with other stakeholders and among themselves. For example, the interactions between patients and health care workers, patients and facilitators, patients and system developers, patients and the knowledge base, and patients and other patient groups were investigated.

The meta-study suggests that there is extensive literature on the perspective of health care workers; however, there is a gap for studying the interaction between patients and other patient groups and, most importantly, the limitation for researching the interaction between system developers and users. In rural areas in low-income economies, when patients are trained to care for other patients, the peer exchange can provide support for counseling and information [20]. Moreover, the interaction with system developers is key to discovering problems early, identifying demands and requirements, and presenting solutions for complex problems [26]. The gap identified in the literature suggests there lacks a design context for system developers to identify the needs and demands of health care workers and patients. Therefore, creating an open-source mHealth platform to collect data in a limited resource setting is needed. The collaborative development approach will create an open-source interface that will encourage telehealth solutions to adopt common standards and solve the data interoperability problem, making it more cost-effective [24,27]. South Africa was used as a case study for analyzing the barriers and facilitators for implementing telehealth solutions in resource-restricted settings. The study was based on the implementation of a hearing screening device in South Africa over two years. The study analyzed the implementation process at four levels: the community level (individuals), the health provider level (health care professionals), the district health system level, and the macro health level for oversight.

The study concluded that need-based innovation improves the possibility of implementation. Meanwhile, changing the device language to a local language would improve the device’s interoperability. Still, having a feedback channel, improving communication with community health care workers, and having protocols to resolve conflicts have been identified as key barriers to implementation. Lacking long-term national-level political support for the program and dissemination channel is also one of the barriers identified. In addition, there is a need to improve health education on hearing and promoting patient mobility on a patient level. Finally, changing patients’ perceived views on the public health system is also important [27].

Compared with the Chinese health care system, where most of the high-quality hospitals are public, and there is heterogeneity in the culture and language amongst the population, the lessons learned from the South Africa health care system include promoting health literacy for patients and health care workers, offering health education regarding the use of telehealth solutions, and building the education and feedback channel of community health workers.

## Methods

### Overview

We used the research methodology inspired by the interactive learning and action (ILA) approach to design the data collection process and the analytical framework developed by Cesuroglu [28] to perform data analysis.

The study conducted by Dijkman et al [29] suggests that value proposition, customer relationships, and partnerships are the most important attributes of the business model for IoT systems. The study conducted by Cesuroglu [28] identified a modified Murray and Frenk’s framework [30] to assess the performance of the health systems. The barriers and incentives for implementing telehealth solutions at a primary level of health care and the hospital level were identified with this framework. By understanding the demand of each stakeholder on a national and local level, the study can serve as a guide to help telehealth solution providers implement their solutions in China.

### Study Design and Data Collection

**Figure 1:**
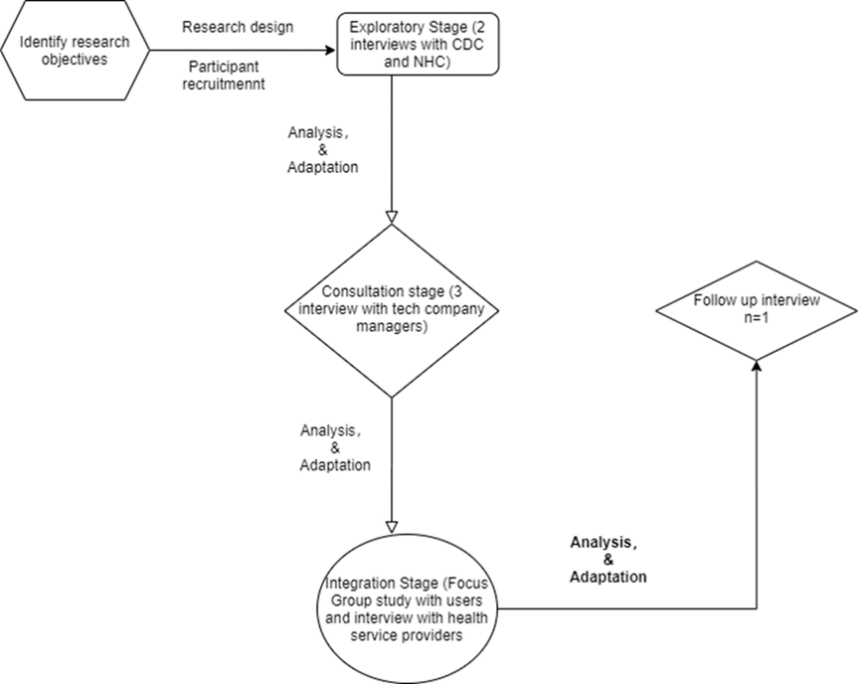
Interactive learning and action process in the research design. CDC: China Center for Disease Control; NHC: National Health Commission.

The study uses an ILA approach to reach patients and health care service providers in the decision-making process. The study covers the three phases of the ILA approach: exploration, consultation, and integration stages (Figure 1).

The first step of empirical analysis is to get insights from stakeholders within the health care system in China. The stakeholder mapping and power analysis is an essential tool to identify the structure of the Chinese health care system. By identifying key stakeholders and their positions, it was plausible to conduct early-stage market surveys regarding the needs and demands of the health care system; moreover, it is key to start with stakeholder analysis when researching business model and market penetration strategies for telehealth solutions in China. Nine stakeholder interview requests were sent with 8 confirmations.

**Figure 2:**
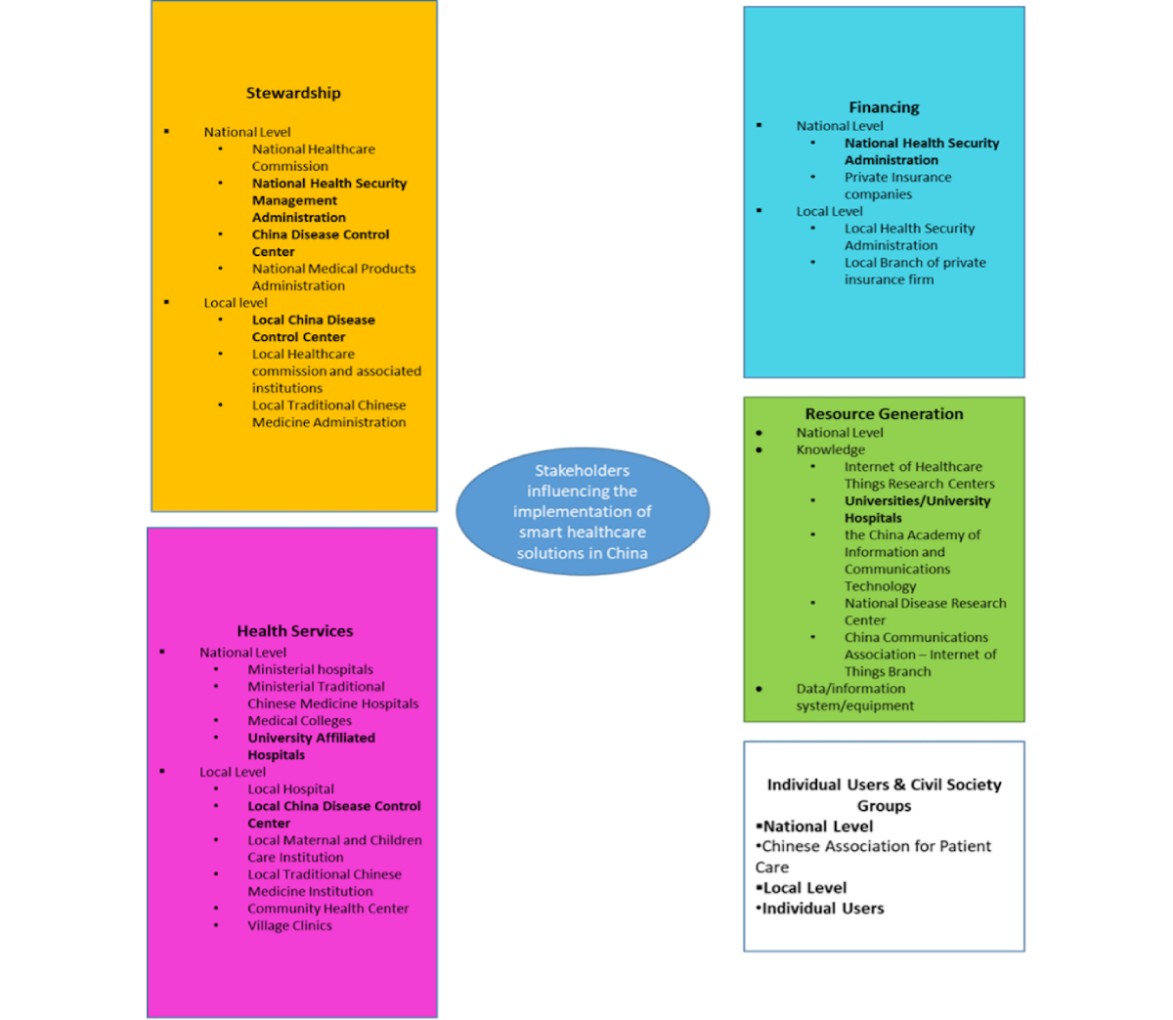
Stakeholder overview for the Chinese health care system.

In terms of selecting semi-structured interviews with policymakers, interviewees among the key actors within the telehealth sector were selected, including representatives from the government (n=2), tech companies (n=6), health service providers (n=2), and individual users (n=64). The list of stakeholders interviewed is presented in Textbox 1. The data collected fits into the stakeholder mapping structure mapped in Figure 2.

**List of stakeholders interviewed in China.**

**Tech companies:**

- Vice President for Wearables (Huawei)
- Director for Investment MIUI (Xiaomi)
- Product Manager for Wearables (Xiaomi)
- Business Manager for Alibaba Cloud ET Medical Brain

**Government officials:**

- Ministry of Human Resources and Social Security (Director for Social Security Pension Fund)
- Interview with the Chronic Disease Management Center Director
- Visited Disease Control Center in Hua Rou, rural Beijing
- Visited Infectious Disease Control Center in Changping

**Doctors/hospitals:**

- Doctor from Cardiovascular Department of Zhongshan Hospital affiliated with Fudan University Medical School
- Doctor from Rehabilitation Department at Hua Shan Hospital affiliated with Fudan University Medical School

### Exploratory Stage

In the exploration stage of the study, interviews (n=2) were carried out with the vice chief of the Basic Medical Insurance Scheme Fund in China and the Director for Chronic Disease Management Unit for the Chinese Centre for Disease Control and Prevention (CDC), respectively.

At the local government level, a brief interview was conducted while visiting the local CDC in Qiaozi county at Huairou district, a rural district of Beijing. By monitoring national pandemics such as SARS, COVID-19, and noncommunicable diseases such as hypertension, diabetes, cardiovascular diseases, etc, the CDC is a key policy advising institution for both the central and local governments [31].

**Figure 3:**
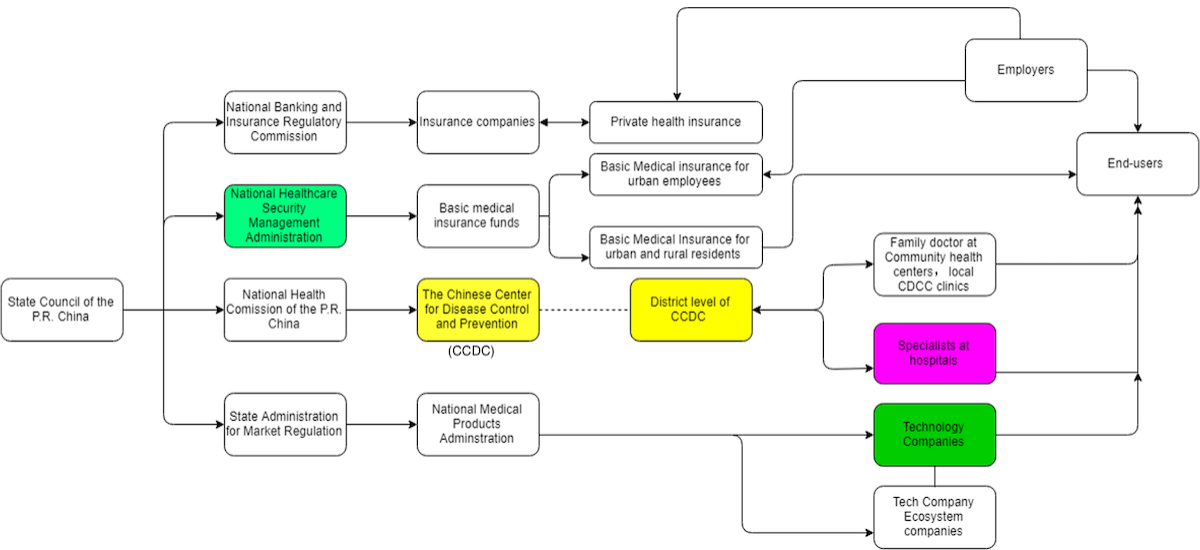
Simplified structure of the Chinese health care system. CCDC: China Center for Disease Control and Prevention.

The basic structure and the position of the CDC in the health care system in China are shown in Figure 2 and Figure 3. As indicated by the graph designed by MENG Qingyue [31], the CDC is the National Health Commission (previously named the “National Health and Family Planning Committee” [NFPC]). It conducts the research and monitoring of communicable and noncommunicable diseases in China. The National Health Commission has replaced the NFPC as the leading health-related policy maker and health insurance administrator. Different provinces and prefectures in China are under the responsibilities of local health commissions. For instance, during COVID-19, the local health commission collected data on new infections, made policy decisions on testing and quarantine, and distributed vaccines to hospitals and community health care centers. By monitoring national pandemics such as SARS, COVID-19, and noncommunicable diseases such as hypertension, diabetes, and cardiovascular diseases, the CDC serves as the key policy advising institution for the central government.

In recent years, a centralized data monitoring system was established where data was reported from local CDC to their supervisors at the local health commission, as showcased in Figure 3. However, this has slowed down the information flow significantly in terms of COVID-19 monitoring and prevented the local CDC in Wuhan from reporting the pandemic directly to the central government. Instead, early COVID-19 cases were reported by hospitals to the local health commission in Wuhan, preventing the implementation of effective measures against the spread of the virus. This suggests a power imbalance between local health care demands and needs and the management of a centralized health data-sharing platform.

### Consultation Stage

In the consultation stage of the study, interviews with wearable and AI business unit managers from Huawei (n=2), Xiaomi (n=3), and Alibaba (n=1) were carried out. Huawei, Xiaomi, and Alibaba were selected as interview subjects because the three companies are the biggest information and communications technology-related solution providers in China. Alibaba was chosen as the leading software solution provider, whereas Huawei and Xiaomi stand out as hardware solution providers. Two interviews were conducted with two business unit leaders in Xiaomi—the Xiaomi MIUI business unit and the Xiaomi wearable business unit. Other tech start-ups follow the lead of Alibaba, Huawei, and Xiaomi. Tech companies dominating the market are often challenged by tech start-ups, with tech giants opting to acquire start-ups challenging their market positions. Alibaba and Tencent have effectively become the most aggressive venture capital funds in China.

**Figure 4:**
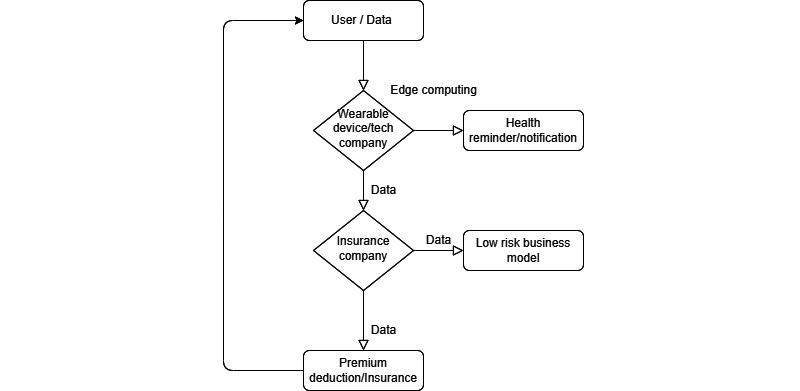
The data-driven business model developed by Huawei. The data collected fits into the mapped stakeholder structure.

For instance, Huawei has developed a data-based driven business model for its wearables and has taken the business model overseas to South Africa, as shown in Figure 4.

**Figure 5:**
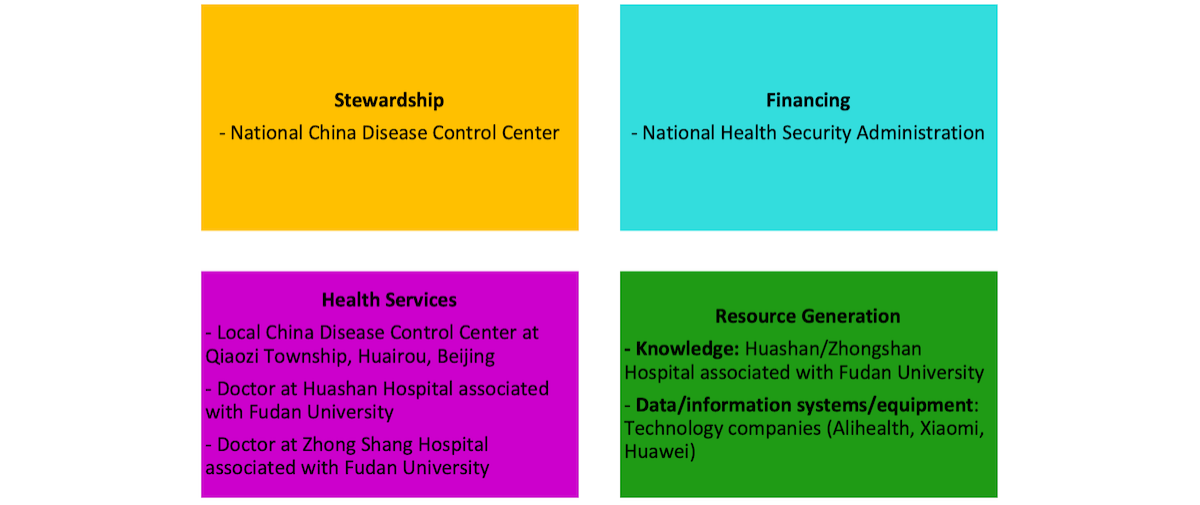
Place of stakeholder (interviewed) in the health care system.

Figure 5 shows a categorized analysis of their roles in the health care system to effectively illustrate the role of each stakeholder interviewed.

In the era of the general data protection regulation (GDPR), the Health Insurance Portability and Accountability Act, and the Personal Information Protection Law of the People's Republic of China, users have the uttermost say in the success of IoT ecosystems. Therefore, this section focuses on presenting factors affecting users’ preference and the use of remote monitoring solutions in four aspects: access to health care, healthy living, elderly care, and chronic disease management.

To explore the attitudes of individual users towards remote monitoring solutions, a focus group study has been conducted in Beijing with support from the research group at the University of China Academy of Science and Beijing Cinso Consulting Co, Ltd. Beijing Cinso Consulting recruited the data subjects, and the study was conducted on the premises of Beijing Cinso Consulting from March 29, 2019, to April 4, 2019.

**Figure 6:**
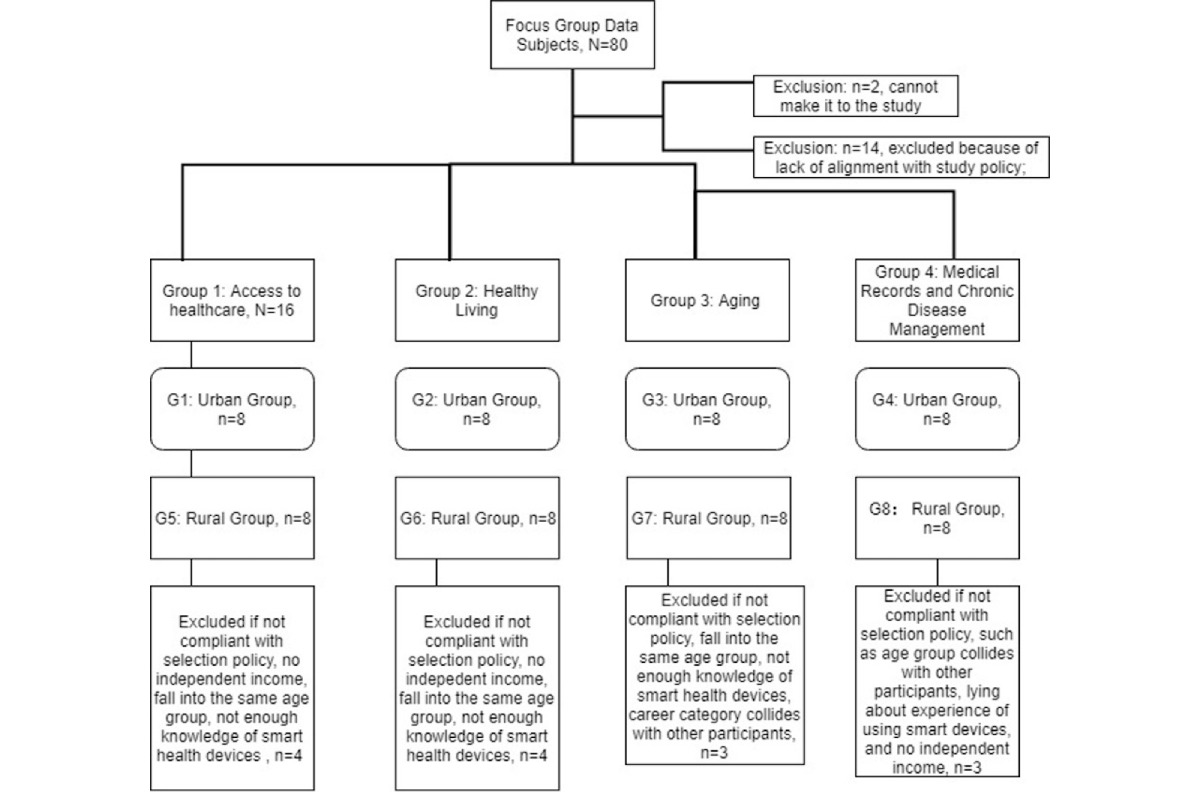
Focus group study data subject selection procedure.

There were 4 groups discussing access to health care, healthy living, elderly care, and chronic disease management. Each group consisted of 16 members, 8 from urban Beijing and 8 from rural Beijing; the focus group data collection process is shown in Figure 6.

### Ethics Approval

The stakeholder interviews did not collect any personal or sensitive data; therefore, no ethics issues were incurred. The questionnaire study and focus group study underwent the ethics procedures at the University of Macerata. Therefore, the Philips Internal Committee Biomedical Experiments does not apply here. Informed consent forms and background information were provided in both English and Chinese.

Following the GDPR requirements on individual data collection, all participants were informed about the purpose of the study, including the scientific research, study procedure, data formats, and processing procedure. The data subjects of the study were informed that the participation was voluntary, and they could leave the study at any time. The participants were also informed that all data would be processed anonymously. Participants were offered RMB 400 (US $62.74) to compensate for their time and transportation. The group discussions were recorded in video and voice, with transcripts taken onsite. All data were stored on an online storage space created by KU Leuven (The Box). Vulnerable groups, such as children and older people (more than 60 years old), were not selected. Some participants in discussion group 3 (elderly care) and group 4 had chronic comorbidities such as obesity, hypertension, or diabetes. Their health conditions were collected to identify whether health factors affect their attitudes towards using remote monitoring solution services and products in different user scenarios. The study has obtained ethics approval from the University of Macerata.

### Stakeholder Power Analysis

Stakeholders can influence and have an impact on the implementation of telehealth solutions. The influence of stakeholders refers to the power of stakeholders in pushing for policies and regulations advantageous for the implementation of telehealth solutions, while the impact of stakeholders refers to the power of stakeholders in promoting the use of certain telehealth solutions. There are three levels of power [32], high-level influence/impact, medium level of influence/impact, and low level of influence/impact.

Bally and Cesuroglu [32] defined the three levels of influence as:

1. Control: The stakeholder has the power to control the implementation of telehealth solutions or stop the integration process.
2. Influence: The stakeholder can influence the integration of telehealth solutions within the health care system. Compared to control level stakeholders, the stakeholder, in this instance, is important but has no decision-making power.
3. Interest/concern. The stakeholder is interested in the use of telehealth solutions but has no significant influence over the integration process.

Each stakeholder's interest, influence, and impact are presented to perform the power analysis for stakeholders related to telehealth solutions in China. Meanwhile, further analysis has been done to understand the attitudes of each stakeholder towards funding, developing, and purchasing internet hospitals services, continuous health management in the home setting services, value-based health care payment systems, the interoperability of health care data services.

## Results

In this section, results from the focus group study within urban Beijing are presented. The stakeholder power mapping framework is used to analyze the facilitators and barriers to implementing telehealth solutions in China.

### Focus Group Study Findings

Table 1 summarizes the findings from the focus group study (group 1: use of telehealth solutions for access to health care services).

Participants indicated their use of telehealth solutions, including smartphones, monitoring devices (cameras and smart audio systems), and wearables. First of all, the telehealth solutions have utility function value, meaning telehealth solutions (devices or services) offer:

1. Convenience by saving time, effort, and money of users.
2. Remote monitoring for users.
3. Environment monitoring functions such as air quality monitoring.

For instance, one participant had installed a smart camera at home to monitor the health condition of her brother, who had experienced a cardiovascular attack. Another participant installed a smart camera at home to monitor the condition of older parents. One participant has a grandmother with hypertension and diabetes; he uses Mi Band to monitor daily steps and sleep quality even though they live together. For those who do not live together, communications happen through WeChat, telephone calls. Some bought smart bands for their parents; however, some older people find it troublesome to use as it needs to be charged all the time.

For smart home devices, some participants resort to smart plugs or switches to control devices at home for safety reasons. For example, some use smart cookers to prepare meals in advance. Some use smart home cameras to monitor their children doing homework. Some use smart floor sweepers for cleaning. When air pollution in Beijing is bad, some use a smart air purifier to monitor air quality and clean the air at home. Some use smart home cameras to talk with pets at home. Some use the camera to monitor people near the door.

There are community projects in rural China where the government offers services checking the health of older people regularly by calling, visiting, conducting medical checkups, and offering lectures for public health purposes.

Most participants do not trust online hospital consultations provided by tech companies such as Alihealth, Ping An Good Doctor, etc. Some participants call the online consultation service with doctors fraudulent, untrustworthy, and profit-originated service. They rely on the services mainly to register at hospitals. For participants residing in rural areas, wait times for seeing doctors at tier-2 and tier-3 hospitals range from half an hour to 2 hours. They also find it difficult to know the specific doctor to reach in the hospital as there is no prior general practitioner consultation. Medical services offered for chronic disease treatment are, in general, expensive and time-consuming.

Long-term relationships between patients and doctors do not exist for almost all participants. Trust between the doctor and patient is low as patients find doctors are unaware of their past problems and unfamiliar with their lifestyles to offer any related advice.

All participants were covered by the basic medical insurance schemes offered by the government, such as Basic Medical Insurance Scheme for Rural Residents, Basic Medical Insurance Scheme for Residents, and Basic Medical Insurance Scheme for Employee. In addition, someome participants have private insurance coverage, which offers additional services such as free ultrasound scans and genetic testing, fast-track tier-3 hospital registration, and family doctor consultation services.

Some participants chose to purchase IoT devices because of the embedded hedonistic value. For instance, some participants buy VR goggles to watch movies and play online games. Some buy smart cameras because they are fun to use. Some have smart audio systems for entertainment during dinner time to prevent kids from watching TV.

Factors affecting buying decisions regarding smart devices include function, price, brand, and convenience of use. For the cost of services, participants report price acceptance between RMB 10-3000 per month (US $1.57-470.56), depending on the service quality offered and whether it is personalized or not. For devices, their price acceptance level ranges from RMB 100 to 30,000 (US $15.69-4705.59), depending largely on the brand. Participants demonstrate a wide knowledge of local brands such as Xiaomi, Huawei, Alihealth, and Chun Yun Doctor and foreign brands such as Philips, Apple, Sony, Samsung, and Siemens.

Participants who own multiple smart home devices are more likely to use telehealth devices such as sports bands. They also tend to exercise regularly and maintain a healthy living style. There is no significant difference between rural and urban residents, given many work in urban areas and live in rural areas in Beijing.

Other factors affecting the use of telehealth devices include trust concerning the data collected from smart devices. For instance, users find the data collected from wearables and smart blood pressure monitors inaccurate or find it impossible to share the data with doctors. This corresponds with interviews where doctors mention they do not trust internet hospital services or the data collected from smart medical devices at home.

Regarding sharing data with insurance companies, about 70% (45/64) of users do not want to share data collected from wearables and smart home devices. They believe insurance companies will surely increase premiums once they find out about their health problems. They also do not trust insurance companies to keep their data safe. However, some are willing to share data with insurance companies for the benefits of accessing a family doctor, free insurance package, reduced/eliminated term payments for wearables, and coverage for expensive diseases such as cancer.

Regarding sharing data with tech companies, most participants acknowledge that tech companies collect their data thoroughly and may share it with third parties. They acknowledge they have no control of the data once it has been collected.

Regarding sharing data with governments, some indicate they want to benefit from the services offered with sharing. Others want to know the purpose of the collection. Most participants want to share health-related data anonymously.

**Table 1:** Summary of the focus group responses on the use of telehealth solutions in accessing health care (urban residents).

	Interviewee 4	Interviewee 1	Interviewee 3	Interviewee 7	Interviewee 2
Private insurance	Yes	No	Yes	No	Yes
Career	TR^a^	NTR^b^	NTR	NTR	NTR
Health awareness	Yes	Yes	Yes	Yes	Yes
Knowledge of telehealth solutions	High	Medium	Not mentioned	Medium	High
Marriage status	MWK^c^	Single	MNK^d^	MWK	MNK
Chronic disease status	Yes	No	Yes	Yes	No
Health status	Suboptimal health status	Suboptimal health status	Suboptimal health status	With chronic disease (diabetes, hypertension)	Suboptimal health status
Health knowledge	High	Medium	Low	Medium	Low
Frequency of the use of telehealth devices	High	Not mentioned	High	Medium	High
Experience in using telehealth devices	Yes, hospital registration (WeChat)	Yes (WeChat)	Yes (WeChat, AI guided patient registration, online consultation)	Yes (WeChat, AI guided patient registration, medication reminder)	Yes (WeChat patient registration, Ping An Online Consultation, Medication reminder)
Community health care/family doctor	Yes	Yes	Yes	Yes	No
Trust of family doctor	High	Not mentioned	High	Low, only to get prescriptions	Low
Prefer online service	Yes	Possible	Yes	Yes	Yes
Pay for online family doctor service	No.	Not mentioned	Yes (prefer service packages or pay for outpatient service)	Yes, pay by the number of times used	Yes, depends on the quality of service
Elderly care service door to door	Yes	Not mentioned	Not mentioned	Not mentioned	No
Prefer service offered, transfer to a specialist at tier-3 hospitals	Yes	Yes	Yes	Not mentioned	Yes
Expected family doctor type	Experienced one’s retired doctors	Save the time for registration with hospitals.	Serving the whole family, offers services such as making an appointment with doctors; pricing mechanism: yearly base plus multiple times payment	Not mentioned	Already use Ping An Good Doctor: pay 199 RMB^e^ for health management package.
Sharing data with the insurance company (trust)	No	Not mentioned	No, I can share with doctors.	No, for privacy reasons, do not wish to disclose medical information with insurance companies; willing to share with family	Yes, the current insurance scheme promotes data sharing with the provider
Age group^f^	3	2	3	5	3
Gender	Female	Male	Female	Male	Female
Household income	High	High	High	High	High
Sharing data with health care providers	Yes (there is no alternative)	Not mentioned	Yes	Not mentioned	Not mentioned
Private insurance coverage	Yes	Not mentioned	Yes	No	Yes

^a^TR: tech-related jobs.

^b^NTR: nontech-related jobs.

^c^MWK: married with kids.

^d^MNK: married, no kids.

^e^A currency exchange rate of ¥1=US $0.16 is applicable.

^f^Proxy for age groups: (2) aged 20-30 years; (3) aged 30-40 years; (4) aged 40-50 years; (5) aged 50-60 years.

### Stakeholder Power Mapping

Among all the stakeholders, except the demand for individual users, the attitudes of institutional stakeholders are important to evaluate as well. In addition, the digitalization of the health care system creates demand from government and health care service providers for telehealth solutions. Before discussing the implementation of specific telehealth solutions, it is thus beneficial to find out the barriers and facilitators for telehealth solutions in China.

Compared with the European telehealth solution implementation process, Chinese medical data are shared more often between different stakeholders. Both European and Chinese governments have national-level digital health development strategies. Before the pandemic, China was leading in providing online medical consultation and prescription services, yet the trust level remained low. During the pandemic, more AI-related image recognition software was fast deployed in China to facilitate COVID-19 diagnosis and treatment. Large-scale data monitoring via mobile phone apps was deployed to track COVID-19 cases and prevent the disease from spreading.

### Stakeholder Identification

Key stakeholders involved in implementing the internet of health care things are categorized into 5 main groups as illustrated in Figure 2:

1. Stewardship group.
2. Financing group.
3. Health services group.
4. Resource generation group.
5. Individual users and civil society groups [32].

Remote health monitoring solutions directly influence groups 3 and 5. Groups 1, 2, and 4 can influence the integration of the internet of health care things solutions. The stakeholder groups can facilitate the development of telehealth solutions by providing infrastructure (such as 5G network), regulation frameworks, financing, and knowledge to help the health care system function.

Among all the stakeholders, 8 semi-structured interviews were conducted to explore attitudes, user experiences, and the needs of stakeholders for telehealth solutions.

### Stakeholder Positioning

The Chinese health care system is a top-down system consisting of several layers: the central government level, the provincial government level, the prefectural level, the county level, and the village level. Top hospitals such as Peking Union Medical College Hospital and China-Japan Friendship Hospital are supervised and financed directly by the National Health Commission. Provincial and prefectural hospitals are supervised and largely financed by the designated provincial and prefectural health commissions. Village clinics are largely self-funded with a small amount of public financial support.

Urban residents spend more on health care than rural residents. Residents in tier-1 cities such as Beijing and Shanghai spend more than the national average on health care services. Meanwhile, local governments finance public health care costs, including funding for hospitals, suggesting developed regions in China have more health care resources than less developed regions in China. The local government and local health commission often have a larger role in providing an immediate response for pandemics such as COVID-19 than the central government. Given the many layers of hierarchy between local and central governments, it may explain why at the beginning of the pandemic, the disease was not recognized as a major public crisis when the first case was reported to the health commission in Wuhan.

Local health security administrations set prices for health care services at public hospitals and decide the coverage of public health insurance schemes. Different regions also have different health insurance coverage and reimbursement standards.

The National Health Commission assumes stewardship function, making health care policies, supervising health service providers, and collecting public health-related data. The National Healthcare Security Management Administration aims to become a strategic buyer of health care services in China and denotes the coverage of public health care insurance schemes. The Basic Employee Medical Insurance Scheme and the Basic Resident Insurance Scheme currently cover 92% of the population in China. Private insurance schemes complement the public health insurance scheme by offering coverage for medical incidents not included in the public health insurance package. Employers can pay for private and public insurance coverage for employees. The State Administration for Market Regulation supervises medical devices, including smart medical devices. Most services tech companies offer land in the lifestyle domain and are not subject to medical device regulation procedures.

To summarize, institutional stakeholders have five types of incentives to implement telehealth solutions.

Firstly, institutional stakeholders implement telehealth solutions because of financial incentives. For instance, companies may wish to improve their market share, promote brand value, enter a new business sector, or promote sales and look for new profit sources. The government may wish to control health care costs.

Secondly, institutional stakeholders may implement telehealth solutions to improve operational efficiency. Health care efficiency may increase by connecting different data sources. Algorithms used to assist health care service providers in making decisions may also improve health care efficiency. Governments may wish to allocate limited financial resources in health care more efficiently.

Thirdly, tech companies may choose to develop telehealth solutions to win government contracts and support. Companies feel the need to answer government policy initiatives for the Internet + Health, Healthy China 2030 Initiative. Companies may also want to participate in smart city initiatives to profit from government contracts and cooperate with local governments for policy support.

Finally, stakeholders may choose to implement telehealth solutions because of special incentives such as health care quality concerns. For example, government agencies may wish to create more employment opportunities. In addition, hospitals and companies may wish to obtain more expertise in AI to improve the health care quality for patients.

### Stakeholder Power Analysis

In the interviews, each representative estimated the power of each stakeholder in implementing telehealth solutions. Three levels of influence were used to estimate the power of each stakeholder.

1. High influence: The stakeholder has the power to control the adaptation of new technology or facilitate such solutions. It also can stop the integration of telehealth solutions.
2. Medium level of influence: The stakeholder plays an important role in the adoption of telehealth solutions but has less control of the process. It can influence the process of adaptation.
3. Low level of influence: The stakeholder is interested in the adoption of telehealth solutions but has little and no significant impacts on adaptation development.

The most relevant quotes from stakeholders’ interviews are presented in Textbox 2.

**Figure 7:**
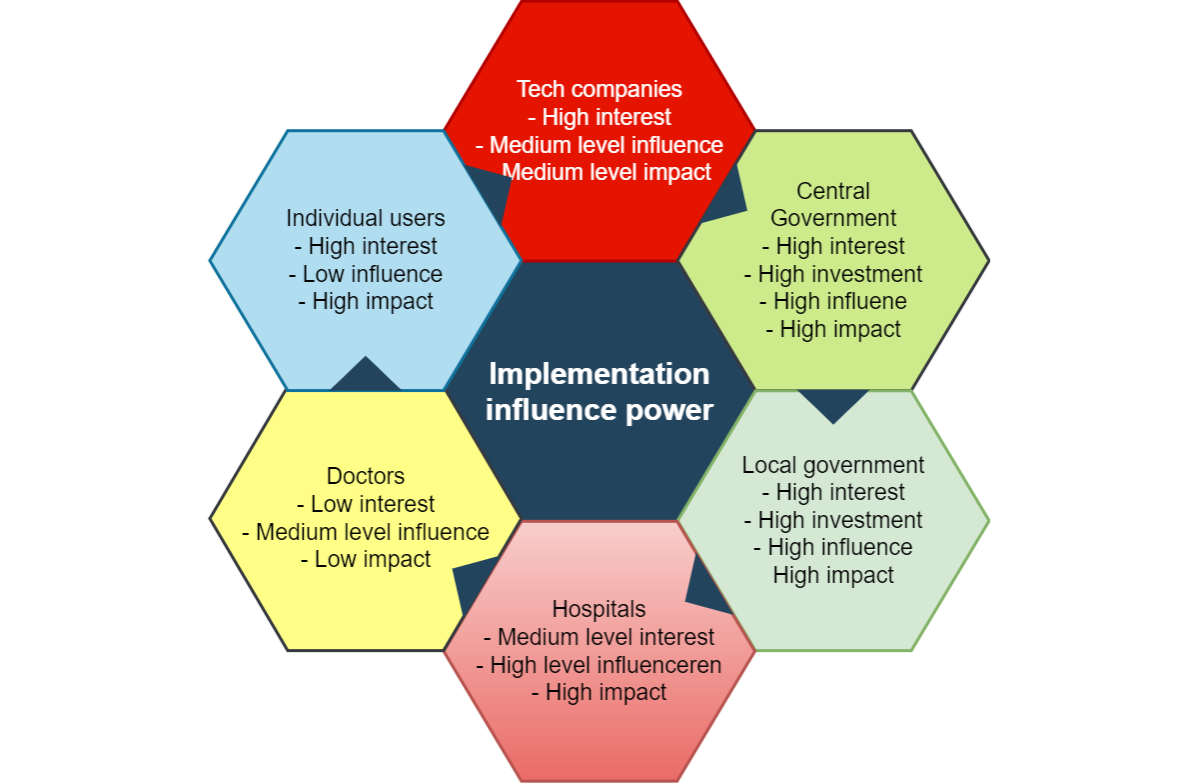
Stakeholder power analysis on the implementation of telehealth solutions.

Figure 7 describes the stakeholder power analysis, including their influence, interest, and the impact they may have in implementing telehealth solutions, such as personalized electronic health records and value-based payment systems for health care services.

**Quotes from stakeholder interviews.**

**Resource Generation (Tech companies):**

“Currently, profits (from telehealth solution platforms) come from online drug sales. This is because, in the short term, it is difficult to transfer users from offline to online to seek healthcare help.”

“It is not an industry-wide approach to integrate all the healthcare-related data of users into a single platform. There is no industrial agglomeration effect.”

“The ownership right of the user’s personal data is not clear…data collected from personal medical devices cannot be shared on third-party platforms.”

**Stewardship/Financing (National Health Commission and China Disease Control Center):**

“The family doctor system and the smart health solutions are in the early stage of development.”

“…the supply side of GP services is small (with 309,000 doctors in China, around 2.2 GPs per 10,000 people at the end of 2018), with little recognition level from the society, and little trust from patients.”

“Seeing a doctor is not as easy as uploading the blood pressure data online. Treating patients demand more communication.”

**Health Services (Hospitals/doctors):**

“From our perspective, the data from medical devices at home settings is very valuable.”

“I would like to consider such data and take it as a reference when I make my diagnosis.”

“The premise for use of data obtained from wearables, except for the convenience provided, is data accuracy. The most important aspect for the use of medical devices in diagnosis is accuracy.”

“The wearable device is at least a few hundred yuan, and the patient may not spend more than ten yuan in the hospital.”

“…the elderly will not use smart medical services. Patients do not trust Internet medical services.”

“The hospital has no motivation to unify the medical record standards.”

Tech companies have a high interest in the integration of telehealth solutions, but they have a medium level of influence and impact. Tech companies train the algorithms with hospitals and doctors and participate in the technical standard-setting process. However, tech companies cannot decide whether or not telehealth solutions can be integrated. Therefore, tech companies have a medium-level influence in standard-setting and a medium level of impact in telehealth solution integration.

Tech companies are subject to strict regulation from the National Medical Products Administration (NMPA) to develop and commercialize telehealth solutions. NMPA is responsible for approving medical devices, in vitro diagnostic solutions, pharmaceutical products, cosmetics, health food, infant formula, and food for special medical purposes. The NMPA promulgated “Medical Device Adverse Event Monitoring and Re-evaluation Management Measures” in August 2018. In 2019, the NMPA issued the “Key points and explanation of deep learning-assisted decision-making medical device software review standards” [33].

When it comes to using AI for diagnosis and triage, tech companies need to obtain level II or level III certificates. Level III devices cover the following categories: active surgical medical devices; passive surgical devices; neuro and cardiovascular surgical instruments; medical imaging equipment; devices for blood transfusion, dialysis, and cardiopulmonary bypass; active implants; passive implants; infusion, recovery, and protective devices; and ophthalmic instruments.

The standards point out that any AI-assisted diagnostic software needs to submit applications regarding the data source, data collection, data processing, and algorithm design and performance, as well as undergo clinical trial and adverse studies submitted to NMPA for approval. In 2020, 9 solutions from 8 companies obtained Level III certificates [33]. These algorithms cover cardiology, neurology, endocrinology, orthopedics, and thoracic surgery. COVID-19 has certainly accelerated the approval process.

From the analysis above, the central government in China can set the standards for the use of AI and IoT devices in the medical setting, while local governments can decide the budget for such solutions. Therefore, both central and local governments have a high influence and impact.

Hospitals care more about reducing misdiagnosis and the prevention of medical accidents. Therefore, they have a medium level of interest in the integration of telehealth solutions. The director of hospitals usually decides whether hospitals will spend on telehealth solutions. Therefore, hospital management has both a high influence and impact regarding the integration of telehealth solutions.

Doctors care about reducing workloads, publishing papers, and getting involved in research projects. They cannot decide whether a hospital can purchase or integrate telehealth solutions. However, top doctors can participate in the policy-making process and have a medium level of influence regarding the formation of policy and standards. Doctors have a low level of influence on integrating processes as they do not have the financial resources to decide the use of such solutions within hospitals.

Individual users are highly interested in using such solutions to save the trouble of going to hospitals or waiting in long queues for treatment. However, they often do not set technical standards or make policies regarding developing telehealth solutions. Yet individual users can decide whether to use telehealth solutions at home; therefore, they have a low level of influence but a high level of impact when it comes to integrating such solutions.

Table 2 illustrates each stakeholder’s position for funding/developing/purchasing specific telehealth solutions such as internet-based hospitals service, personalized/continuous electronic health care records (EHR) service, family doctor service for chronic disease management, etc.

**Table 2:** Stakeholder position on adoption of telehealth solutions.

Key points of view	Tech companies	Doctors	Government organizations	Consumers
General attitudes about smart health solutions	Positive	Positive	Neutral/negative	Neutral
Interact with patients via internet-based hospitals	Positive	Negative	Negative	Negative
Personalized electronic health records	Positive	Neutral	Positive	Neutral
Family doctor service for chronic disease management	Negative	Negative	Positive	Positive
Continuous health monitoring with home devices	Negative	Negative	Positive	Negative
Share health-related data with insurance companies	Negative	Negative	Neutral	Negative
Using public medical insurance to pay for smart health solutions	Positive	Neutral	Negative	Positive
Using private medical insurance to pay for smart health solutions	Negative	Neutral	Negative	Negative
Value-based health care payment schemes	Neutral	Negative	Positive	Positive
Separate approval process for medical use of wearable devices	Positive	Positive/neutral	Negative	Neutral
Interoperability of hospital information system	Positive	Positive/neutral	Negative	Positive

The stakeholders point out the challenges for realizing telehealth solutions in China.

First, strict regulations for medical use lead to difficulties in the commercialization of the health-related functions for wearables. Relevant approval usually takes 5 to 10 years, depending on the country and specific function submitted for approval. Some of the monitoring methods, like the pumping in blood pressure monitoring, disturb users’ sleep, and therefore, cannot be used for 24/7 monitoring. However, this is the only acceptable method for medical device regulators. However, new ways of monitoring blood pressure via photoplethysmogram cannot be commercialized due to strict regulations on medical use of biometrics data monitoring.

Second, most doctors do not make decisions based on data collected via wearables or home medical devices for diagnosis or treatment. Currently, wearables and home medical devices are not connected with the hospital EHR system; therefore, doctors only use data collected by patients at home for reference.

Third, there are no national and regional level health care data-sharing platforms.

Fourthly, the social security system does not cover the cost of wearables. There are no standards for evaluating the effectiveness of wearable devices for chronic disease management on a population level. It is also difficult for patients to be reimbursed by the public insurance system.

Fifthly, consumers often find it untrustworthy to share biometrics data with different stakeholders. Users are not willing to share their data, whether it is with doctors, insurance providers, or family and friends. Most people, however, do not know how the data collected on wearables are stored, shared, and used; data can flow to Facebook, Apple, Google, Baidu, and Amazon without users’ knowledge, let alone consent. This leads to a monopoly in tech companies with regard to data storage and processing. These companies already have the most robust computing power and storage units and the best algorithms.

Lastly, there is no clear legal definition of the ownership of personal data. Users may find they have lost the rights to their data-to-data controllers easily. Given the wide industrial approach of uploading and processing data on the cloud, it is almost impossible to track data flow once it leaves the device.

## Discussion

To fully implement IoT solutions within the health care industry, health care service providers need to work with government officials to build up data-sharing platforms, eliminating duplicated procedures and facilitating access to medical records. Medical device regulators shall adopt the technology standards along with technology development.

Local authorities shall be given more authority to test the health care programs based on their priorities and get involved with retired populations that may return to the workforce. Employers may find older people more patient, careful, trained, and trustworthy than imagined. In this way, health care quality variation can be adjusted, and optimal pathways can be promoted. In addition, when it comes to new drugs and new technology approvals, population health assessments based on big data will make it possible for more policymakers to say yes or no to new drugs and treatment methodologies with much more efficiency.

Rising health care costs associated with the aging population have led to concerns that the retired and older people may inflict great stress on the welfare system. Wearable devices may make it possible to monitor the health conditions of older people and allow them to live independently for as long as possible. By interacting with technology and initiating data sharing, the life quality of older people may also improve. There are barriers to data interoperability, technology standards, and privacy and safety concerns involved with the medical use of IoT devices. Policymakers may need to follow up more closely with technological development to adapt the technology usage standards and improve public awareness about the data storage, usage, and sharing involved in wearable technology and AI for medical use. A clear definition of data ownership would also help determine the ethical and legal methods of personal biometrical data collection in the coming era of the internet of health care things.

## Abbreviations

AI: artificial intelligence
CDC: Chinese Centre for Disease Control and Prevention
EHR: electronic health care records
GDPR: general data protection regulation
ILA: interactive learning and action
IoT: internet of things
mHealth: mobile health
NFPC: National Health and Family Planning Committee
NMPA: National Medical Products Administration
VR: virtual reality
